# A monthly quality assurance procedure for 3D surface imaging

**DOI:** 10.1120/jacmp.v12i1.3338

**Published:** 2010-12-21

**Authors:** H. Omar Wooten, Eric E. Klein, Garima Gokrhoo, Lakshmi Santanam

**Affiliations:** ^1^ Department of Radiation Oncology University of California Davis, Sacramento CA 95817 USA; ^2^ Department of Radiation Oncology Washington University in St. Louis St. Louis MI 63110 USA

**Keywords:** surface imaging, localization, quality assurance, radiotherapy

## Abstract

A procedure for periodic quality assurance of a video surface imaging system is introduced. AlignRT is a video camera‐based patient localization system that captures and compares images of a patient's topography to a DICOM‐formatted external contour, then calculates shifts required to accurately reposition the patient. This technical note describes the tools and methods implemented in our department to verify correct and accurate operation of the AlignRT hardware and software components. The procedure described is performed monthly and complements a daily calibration of the system.

PACS number: 87.56Fc

## I. INTRODUCTION

AlignRT (Vision RT, London, UK) is a commercial patient localization and tracking system for image‐guided radiation therapy (IGRT). It uses a CT‐derived surface image as a reference and 3D representations of the patient's topography obtained from a video camera‐based in‐room monitoring system.^(^ ^1^ ^)^ The system is capable of: (i) importing DICOM‐formatted external surface structures from commercial treatment planning systems (TPS), (ii) direct connectivity with certain linear accelerator treatment couches for performing shifts automatically, and (iii) tracking the patient's surface in real time. The AlignRT system does not require surface fiducials, nor does it require irradiation of the patient. The system has a resolution of 1024 by 768 and can capture as many as 20,000 points for comparison. Two projector/camera systems are the key components of the system. Two high resolution optical cameras capture an image as the projectors illuminate a speckle pattern onto the patient.

Our department has installed the AlignRT system in two treatment rooms occupied by Elekta and Varian linear accelerators. The department has established procedures and protocols primarily for breast patients being treated with tangential photon and en face electron beams. This article describes the monthly procedure and tools developed in our department and procedures to monitor the constancy and performance of the AlignRT systems. These procedures have been implemented as part of the overall quality assurance program in our department.

The AlignRT system has been a successful alternative patient localization tool for patients receiving conventional breast and accelerated partial breast radiation therapy.^(^ ^2^ ^,^ ^3^ ^)^ This technical note focuses on quality assurance procedures developed for the static image acquisition mode. In our department, AlignRT is used to capture a static image of the patient once the patient is aligned with the machine isocenter by lasers and an optical distance indicator. Then, based on the position of a surface region relative to the same region on an external surface contour, AlignRT calculates couch shifts in the lateral, longitudinal and vertical directions. The monthly QA procedures were developed in part to test the accuracy of these calculated shifts.

## II. MATERIALS AND METHODS

**Figure 1:**
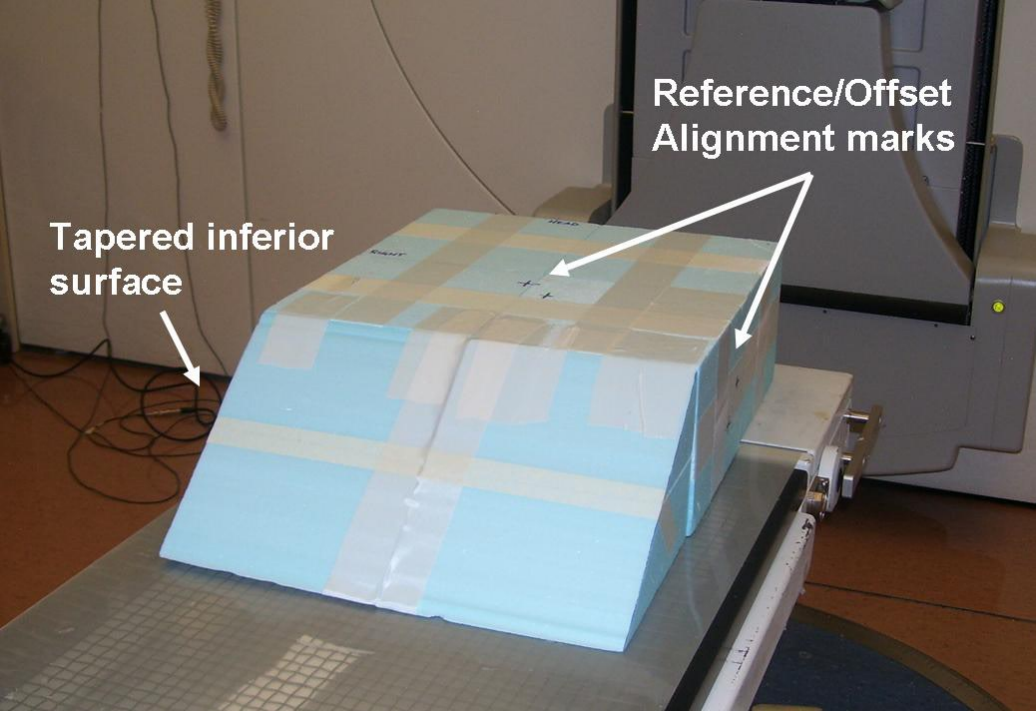
Monthly QA phantom. This phantom is constructed with blocks of styrofoam joined with tape. Color‐coded isocenter alignment markings are etched on anterior and lateral surfaces indicating both “reference” and “offset” positions.

A prototype AlignRT monthly phantom is constructed of styrofoam blocks (Fig. 1). The inferior end of the phantom is tapered such that it provides an adequate surface from which the speckled light pattern from AlignRT may reflect. The phantom has color‐coded marks indicating central axis (anterior surface) and laser (lateral surfaces) alignment marks used to align the phantom with the machine isocenter. One color represents alignment marks when the phantom is in the “reference” position, and the other color indicates an “offset” position, whereby the phantom is shifted 3 cm in each of the lateral, longitudinal and vertical directions. This phantom is placed on top of the treatment couch, and set up to isocenter using the reference marks.

**Figure 2:**
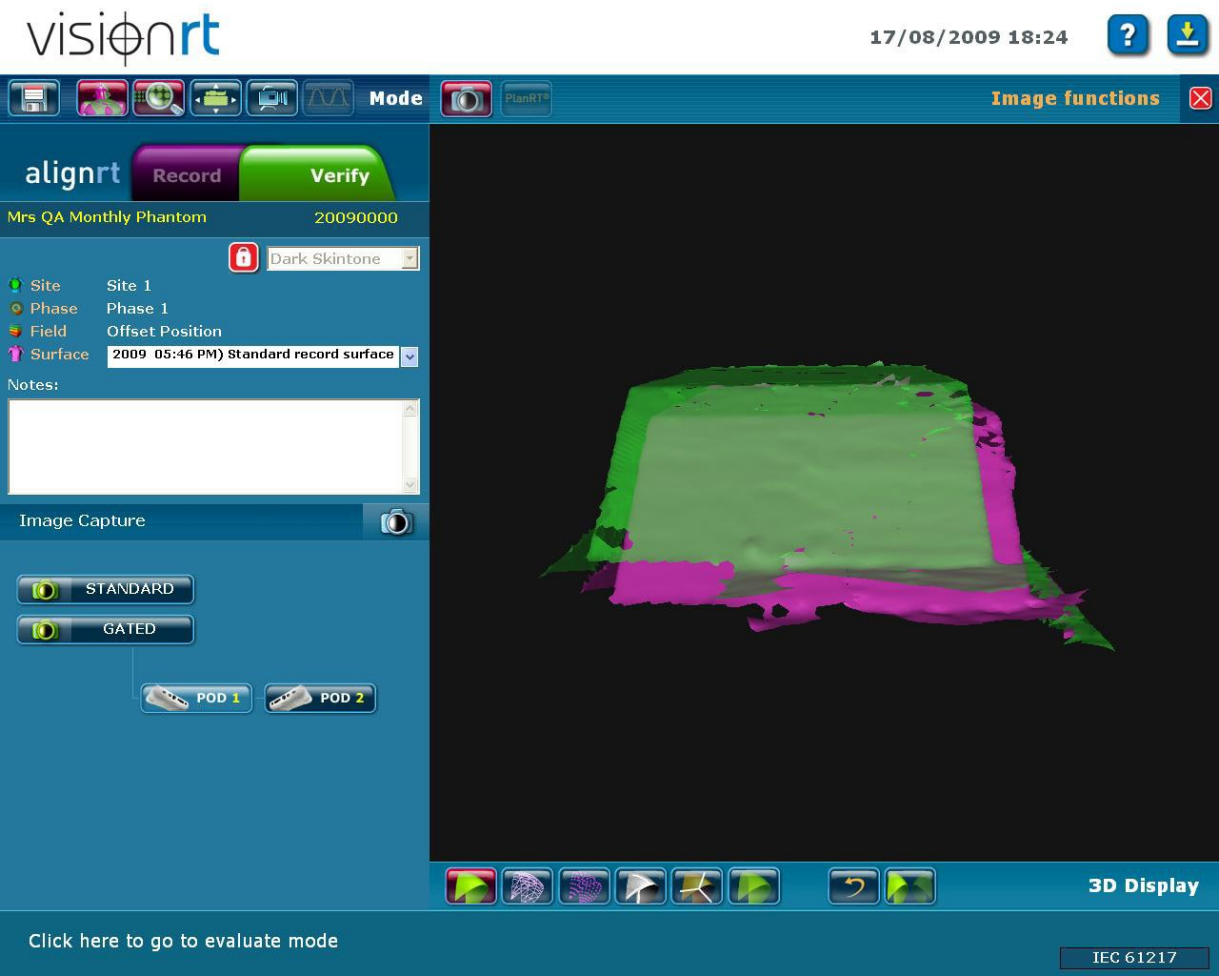
Overlay of capture monthly (green) and reference (pink) phantom surfaces when the phantom is set up to offset marks.

**Figure 3:**
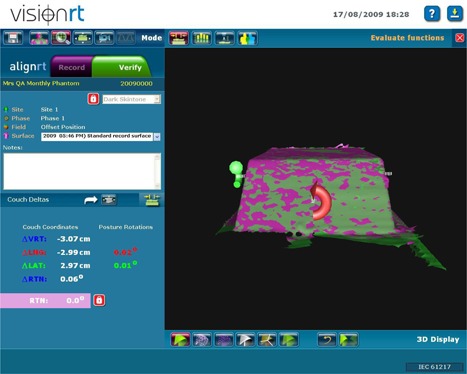
Couch shifts determined by the AlignRT system (cm): −3.07 vertical, −2.99 longitudinally, and 2.97 laterally.

**Figure 4:**
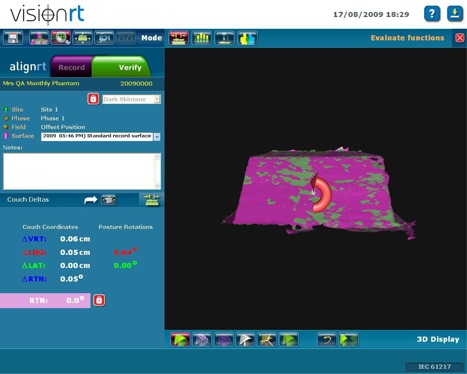
Post‐shift image and calculated shifts (cm): 0.06 vertically, 0.05 longitudinally, and 0.0 laterally.

A reference image is then obtained using the AlignRT system. This image becomes the standard to which future comparisons are made. Each month, a test image is obtained with the phantom aligned to isocenter using the offset markings (Fig. 2). The AlignRT software calculates the shift required to return the phantom to the reference position (Fig. 3). We record the actual shift calculated by the system and compare the values against the 3.0 cm values expected for all three directions (Table 1). The couch shifts are then applied, and another image is obtained. Shifts are then recalculated and compared to the 0.0 cm values expected for all three directions (Fig. 4), recorded (Table 2) and compared to our current tolerance of ±0.2 cm. This tolerance has shown to be acceptable and repeatedly achievable, as the system provides sufficient resolution and precision. As some of our breast RT techniques include partial breast irradiation (PBI) where high doses per fraction are prescribed (4 Gy), an accuracy of 0.2 cm is necessary. After the shifts are recalculated, the field central axis and lateral lasers should intersect the color coded reference marks on the phantom. If they do not, further investigation is required, as the lasers or field light may be out of alignment.

**Table 1:** A table for recording shifts as determined by AlignRT after setting up the monthly phantom to isocenter using the offset marks.

*Direction*	*Expected (cm)*	*Actual (cm)*	*Tolerance*	*Within Tolerance? (Y/N)*
Vertical	3.0		±2 mm	
Longitudinal	3.0		±2 mm	
Lateral	3.0		±2 mm	

**Table 2:** A table for recording shifts as determined by AlignRT after applying the shifts.

*Direction*	*Expected (cm)*	*Actual (cm)*	*Tolerance*	*Within Tolerance? (Y/N)*
Vertical	0.0		±2 mm	
Longitudinal	0.0		±2 mm	
Lateral	0.0		±2 mm	

## III. DISCUSSION

Bert et al.^(^ ^3^ ^)^ demonstrated that breast surface congruence is increased when 3D surface localization is employed for partial breast irradiation. AlignRT is therefore a useful clinical tool and its use will likely increase over time. As with other technologies introduced into the clinic, in addition to the initial evaluation and acceptance testing, routine evaluation provides an additional level of patient safety. The correct operation of new technologies is even more important for dose escalation trials and accelerated treatment protocols. This note provides a simple method of ensuring that the AlignRT system as a whole (cameras and algorithms) are working as expected.

This procedure is quite similar to daily quality assurance tests performed on other systems such as on‐board imaging devices. The AlignRT system is much less complicated and uses no moving components. In our clinic, we have implemented a daily QA procedure that tests the overall alignment of the AlignRT by using a calibration plate. The Daily QA test consists of placing a calibration plate with an array of black dots on the couch at the isocenter plane. A daily image of the plate is captured, from which the AlignRT algorithm determines the average deviation of the dots with respect to their expected positions. We believe this is a suitable test prior to daily use, and we recommend performing this procedure immediately prior to beginning the monthly tests to identify any potential underlying problems with the camera alignment. Any potential problems with the algorithms, software functionality, or couch motion will then be identified using the monthly QA phantom. If the monthly tolerance of 0.2 cm is exceeded but the shift is less than 0.3 cm, treatments may continue until time allows for a calibration to be performed, usually requiring 30–40 minutes. If the tolerance exceeds 0.3 cm, recalibration of the system is required prior to clinical use.

## IV. CONCLUSIONS

We have conducted the monthly review for our two AlignRT systems for almost 1 year. The procedure takes about 15 minutes, and both pre‐shift and post‐shift calculations determined by AlignRT have been reproducible within the 0.2 cm tolerance. As previously mentioned, the existing phantom is a prototype, and we recognize that this procedure will benefit from a phantom constructed of more rigid and sturdy material as well as precisely etched markings for aligning the phantom with the room lasers. We therefore intend to replace the existing phantom soon. An additional benefit of the monthly review is to ensure the software has not become corrupted. The monthly procedure will also be performed if a system recalibration occurs, if a system problem is detected, or after the system is serviced. We are now developing annual QA procedures that will extend the monthly procedure over a wider detection range, and verify accurate performance of AlignRT system tools such as the surface statistical analysis functions.
